# Agency as power: An ecological exploration of an emerging language teacher leaders’ emotional changes in an educational reform

**DOI:** 10.3389/fpsyg.2022.958260

**Published:** 2022-09-09

**Authors:** Yuan Gao, Yaqiong Cui

**Affiliations:** Department of Foreign Languages, University of Chinese Academy of Sciences, Beijing, China

**Keywords:** language teacher emotion, teacher leader, educational change, ecological perspective, language teacher psychology

## Abstract

Teacher emotion, an important aspect of language teacher psychology (LTP), has recently drawn growing attention in language teacher development studies. Previous research has shown that language teachers, typically pressured by heavy workloads, may face emotional challenges from multiplied sources, especially in the context of educational changes such as curriculum reform and the COVID-19 emergency. Current literature on teachers’ emotions largely centers around ordinary language teachers, with teacher leaders whose agentic actions often exert greater influence on the effectiveness of educational changes rarely examined. Situated in a top-tier research university that has been promoting an English for Academic Purposes reform to enhance its science students’ multilingual competence in academic contexts, this longitudinal case study tracked the emotional trajectory of an English teacher, Lea, for 5 years. Adopting an ecological perspective, our study confirms that language teachers’ emotions vary across the reform ecosystems and extends the current inquiry by conceptualizing the intricately interrelated teacher emotion, agency, power, and identity as dynamic constructs. This study also reveals how the reform-inflected emotional changes were associated with Lea’s EAP teacher and teacher leader identity construction, with both identities reinforcing each other, which to some extent reconciled Lea’s emotional tensions. Our study bears significant implications for language teachers involved in educational reform, teacher leaders, and school administrators.

## Introduction

Over the past two decades, teacher emotions as an important aspect of language teacher psychology (LTP) have drawn extensive attention in language teacher development studies (Jiang et al., 2021). Previous research has shown that language teachers, typically pressured by heavy workloads (MacIntyre et al., 2019), may face increased sources of emotional challenges (Horwitz, 1996) and need to make a range of emotional investments (Loh and Liew, 2016), especially in the context of educational changes such as curriculum reform (e.g., Campion, 2016; Gao, 2018; Gao and Cui, 2021a,b) and the COVID-19 emergency (MacIntyre et al., 2020). Current literature on teacher emotions largely centers around ordinary language teachers, with teacher leaders whose agentic actions often exert great influence on the effectiveness of educational changes (Harris and Muijs, 2003; Fullan, 2005) rarely examined. In particular, no research efforts have been made to explore the emotional experiences of emerging language teacher leaders who are evolving into their leadership identities (Baecher, 2012) while navigating the complex power relations of multiple stakeholders in the emotionally demanding educational reforms.

A recent critical approach to teacher emotions (e.g., Benesch, 2012) has focused on the intersection of teacher emotions with power relations. Unfavorable institutional power relations may cause conflicts in teachers’ beliefs and values, thus resulting in teachers’ emotional dissonance (Benesch, 2017) in the workplace. Similarly, Zembylas (2004) revealed how power relations may influence workplace values and discourses, and hence teachers’ experienced emotions. Teacher emotions are likewise conceptualized by Zembylas (2005) as “continually constructed and reproduced through interactions of domination and resistance” (p. 59). The critical approach brings to attention how the power exercised in the workplace may have a “central” (Benesch, 2018, p. 60) role in shaping teachers’ emotional experiences and how emotions can be theorized as potential agency and source of engagement that could guide teachers for effective actions (Benesch, 2018) to contest such workplace power relations. However, previous research has mainly examined the unidirectional influence of established power relations upon teachers’ emotional responses and their consequent agentic actions, without considering how teachers’ emotional reactions may help build workplace power relations, and particularly develop emerging leaders in a constantly changing work environment. The present longitudinal study, therefore, aims to explore how an emerging language teacher leader’s emotional experiences over an educational reform may empower her agency (Taylor et al., 2020) and may establish her identity of power as a teacher leader. Our study may bear implications for educational reform participating teachers, teacher leaders, and school administrators.

### Identity construction in the reform context

The post-structuralist perspective has viewed teacher identity as an ongoing co-constructive process in situated social contexts (Thomas and Beauchamp, 2011; Taylor, 2017; Tao and Gao, 2018). According to Norton (2013), identity is defined as “the way a person understands his or her relationship to the world, how that relationship is structured across time and space, and how the person understands possibilities for the future” (p. 4). Teacher identity, therefore, develops in a teacher’s search for the sense of self among multiple identities (Friesen and Besley, 2013) as s/he navigates pathways to the future amid the changing context (Barcelos, 2015).

Educational reform, as one example of such changing context, however, can be an “identity-disrupting” (Kennedy, 2020, p. 58) moment when teachers’ long-held set of harmonious identities (Beijaard et al., 2004) may become incoherent and even conflicting with their reform-required new identities. Such identity discrepancies (Markus and Nurius, 1986; Higgins, 1987) may force teachers to re-interpret their own values and their past teaching experiences (Flores and Day, 2006) as they struggle to forge their future new teacher identities, which may give rise to immense emotional tensions (Arvaja, 2018). For emerging teacher leaders in reform, it could be even more difficult to maintain identity stability and continuity because their dynamic equilibrium of identities and emotions (Taylor, 1989) may be seriously disturbed by both their changing teacher role and their developing leader role in the reform community. In short, the instability and discontinuity of teacher identity (Akkerman and Meijer, 2011; Ruohotie-Lyhty, 2013), especially in an educational reform context, may see teachers’ past and future identities eventually synthesizing to realize “identity achievement” (Friesen and Besley, 2013, p. 24), including a leadership identity. However, there is a dearth of literature on emerging teacher leaders’ identity development in the reform context, making it necessary to investigate how emerging teacher leaders achieve their leadership identity and gain power position in the reform community.

### Power construction in the reform community

The post-structuralist perspective also adds the idea of power to the understanding of identity (Ruohotie-Lyhty et al., 2021). Following Foucault’s (1972) tradition, power is co-constructed in dynamic and fluid social relations. The socially constructed teacher identities are thus inevitably intertwined with power negotiations (Chamberlain et al., 2020), making identities “a site of struggle” (Norton and Toohey, 2011, p. 414) where there are often competing narratives of power (Norton, 2013) that are produced by individuals either of discrepant selves (Higgins, 1987) or in different power positions.

A teacher leader has traditionally been constructed as an identity of power, often generating power inequalities (Shi and Yang, 2014) between teachers and teacher leaders. Negotiating such power inequalities may imply the possibility of resistance (To, 2006) that might lead to the change of power relations, especially in a highly fluid educational reform reality. In a reform community where the sense of centripetal connection (Lave and Wenger, 1991) with the central power position is yet achieved (van Lankveld et al., 2017), reform leaders may be seriously challenged particularly when they are similar in short of reform-related insider competence as other community members. For example, although the leading power and authoritative role of reform leaders are usually linked with their professional knowledge and expertise (Adey, 2000; Gray and Morton, 2018), many reform leaders in reality may find themselves inadequately equipped with the reform-required knowledge because a highly demanding educational reform is usually a journey into the unknown (Campion, 2016; Gao, 2018) where reform participants may feel disoriented and unsure (e.g., Gao and Cui, 2021a). This makes reform leaders’ development of their leading power in the reform context a topic worthy of in-depth exploration, especially for emerging leaders whose lack of leadership experience may particularly disadvantage themselves in an educational change.

### Individual agency and relational agency

The agentic role of teachers in conjunction with their identity construction and emotional changes has been typically viewed in their responses to educational changes (Tao and Gao, 2017; Imants and van der Wal, 2020). Viewed as a teacher’s conscious efforts to “resist feelings of powerlessness” (Ollerhead, 2010, p. 607) and “active efforts to make choices and intentional action in a way that makes a significant difference” (Toom et al., 2015, p. 615), the notion of teacher agency stresses teachers as active agents who act with independence and in creative ways (Insulander et al., 2019). In educational reform, individual teachers have the capacity to respond strategically to the reform (Dover et al., 2016), control their daily practices (Ketelaar et al., 2012), make choices and decisions (Pyhältö et al., 2012), and take intentional actions toward the effective outcomes they desire (Ruan et al., 2020). Though often conceived of as an individual characteristic (Huy et al., 2016) and a personal effort, teacher agency is also addressed at the relational and social level (Edwards, 2005; Vitanova, 2018), which often results from the mutual interplay and reciprocal relationship between individuals as well as individuals and their situated contexts (Leijen et al., 2020). According to Edwards (2009), relational agency refers to “a capacity to align one’s thought and actions with those of others in order to interpret problems of practice and to respond to those interpretations” (p. 197). As a “relationally” (Vähäsantanen et al., 2009, p. 395) and “continually” (Priestly et al., 2012, p. 197) emergent process to develop collaborations between individuals (Wright, 2015), a relational agency may not be immediately achieved in an educational reform, because individual teachers may agentively “resist” (Ollerhead, 2010, p. 607) the reform requirements (Wang et al., 2017) and are reluctant to conform to the reform innovations (Sannino, 2010) promoted by their community leader.

A reform leader’s individual agency and reform participants’ relational agency are in a complex and usually uneasy relationship (Gurney and Liyanage, 2016) due to the unsettled power relations in a challenging reform context; the misalignment between the individual and relational agency within a reform community may generate emotional tensions and produce power challenges to the reform leader. To guarantee the success of the reform, a reform leader needs to exercise the power to involve the reform community members in active efforts to make changes (Sannino, 2010) and enable the members’ collaboration and capacity to act in agreement with the reform goals (Wright, 2015). Such efforts, in turn, may enable the community leader to “gain[s] authority by being recognized by the[a] community and receiving support from the[a] community” (Engeström, 2009, p. 317). However, how an emerging teacher leader fosters power and affords agency in the possibly incompatible relationships of individual agency and relational agency in a reform community is basically unknown, and how power and agency may interact to develop teacher leader identity is never explored, thus calling for research attention to examine their relations in educational reforms.

### Teacher emotions in reform ecology

Educational reform is a complex emotional arena (Sachs and Blackmore, 1998) where teachers constantly engage in emotional interactions with students, colleagues, parents, reform leaders, school administrators, and others (Cowie, 2011; Yin and Lee, 2012; Chen, 2016) involved in the reform ecology. Theorized as a relational rather than a pure personal phenomenon in recent literature (Zembylas, 2002), teacher emotions are viewed as socio-culturally constructed and contextualized in specific educational settings (López and Cárdenas, 2014; Barcelos, 2015) where multiple stakeholders may form various power relations with one another.

Teacher emotions are dynamic in nature due to individuals’ “multilayered” (Hsieh et al., 2022, p. 196) identities in emotional ecology (Zembylas, 2007). The complexity of individual teachers’ emotional world can be examined by Bronfenbrenner’s (2005) ecological system framework which considers teachers as situated within a multilayered system of relationships among nested environments (Cross and Hong, 2012; Chen, 2017): microsystem, mesosystem, exosystem, macrosystem, and chronosystem. The microsystem comprises the innermost layer of the ecology where teachers emotionally relate themselves to students, families, and friends. The mesosystem contains the emotional transactions of teachers with their colleagues and school administrators. The exosystem further extends to larger social contexts where teachers may form emotional connections with students’ parents and organizations. The macrosystem constitutes the overarching institutions at the societal level in which teachers interact with the values, norms, laws, and customs of a particular culture. The chronosystem concerns the factor of time since the relationship between individual teachers and their environment may change as time passes. As teacher emotions may vary in different ecosystems, the multilayered workplace ecology may also shape power dynamics at work, with power positions differing across ecosystems and influencing each other. For example, a teacher leader’s power interaction with the university in the exosystem may hierarchically (Darling-Hammond et al., 1995) influence leadership practices in the inner-level mesosystem, and the leading experience with colleagues in the mesosystem may support the power position (Zhang and Henderson, 2018) in the outer-level exosystem.

Unlike the micro-, meso-, and exo-systems which encompass interactions among people, the macro- and chrono-systems that involve abstract social entities and time may bear significant influence on and bring considerable changes to power and emotion in other ecosystems. The social values represented in the macrosystem usually structure the power relations and emotional experiences in the micro-, meso-, and exo-systems, making power and emotion culture-specific phenomena (Yeung, 2000; Elfenbein and Ambaby, 2002; Wu and Chen, 2018). For instance, the Chinese culture values collectivist social harmony and power hierarchy (Hofstede, 2011; Sundararajan, 2015), which may characterize the emotion and power experienced in the inner systems; however, an educational reform may make it difficult to maintain the emotional harmony and power hierarchy, creating conflicts across ecosystems, which may call for individuals’ agentic actions to strike a balance in the dynamics. Emotion and power within and across ecosystems may also change with time. However, substantial gaps remain in our understanding of how emotion and power develop over time, in particular, in a long-time reform process, and about how the identity of power emerges from emotional challenges and agentic actions. Therefore, the present study aims to answer the following research questions:

(1) How do emerging teacher leaders’ emotions change in reform ecology? And how do their emotional changes relate to their agency?
(2) How do emerging teacher leaders’ agentic actions help build their leadership identity?
(3) How does the development of teacher leader identity change emerging teacher leaders’ emotional experiences?

## Methodology

### Context and participant

China’s Ministry of Education launched an educational reform in 2017, known as the “Double First-Class” initiative, aiming to build a group of world elite universities and disciplines by the end of 2050. This nationwide reform calls for extensive changes in English teaching which is given a “new mission, higher demands, and broader scope” (Ministry of Education., 2017). As a consequence, English teachers in many Chinese universities have been encouraged to redesign their curriculum for English for General Purposes (EGP) and offer English for Academic Purposes (EAP) or English for Specific Purposes (ESP) courses that are discipline-related and content-focused.

The present case study was situated in a research university in Beijing, home to nearly 2,000 undergraduates and over 50,000 graduate students. With its educational goal to train future scientists, this university highly welcomes the EAP curriculum reform. This inquiry is part of a longitudinal research project from 2017 to explore Chinese EFL teachers’ professional development in the EAP reform. Thirteen teachers from the EAP reform team participated in the project. Their background varied in terms of professional rank (lecturer, associate professor, and full professor), education (MA, Ph.D. candidate, and Ph.D.), research interest (linguistics, literature, and translation and interpretation), gender (male and female), and years of teaching (1–24 years), which roughly depicts the English teacher population in higher education institutions in China. Two teachers withdrew in the mid and end of 2018, respectively.

The EAP reform community was formed in January 2017 by members of diverse backgrounds. They were recruited by the team leader and were allowed to leave the team at any time. To advance the EAP reform gradually, the reform team has been actively involved in a variety of tasks, including compiling a reading-and-writing-focused EAP textbook for science graduate students, teaching EAP courses, developing localized EAP tests, and making an EAP textbook-based massive open online course (MOOC).

Lea (a pseudonym), the reform leader, is the focal participant in this study. Before joining and leading the reform community, she had taught EGP for more than 20 years without any EAP teaching experience. Despite her past position as an assistant to the EGP team leader, she had no previous leading experience on her own. She was assigned the leader role because she had been aware of the academic needs of her science students and took some individual initiatives to make the EGP-EAP change. Lea was chosen as the focal participant for the following reasons. First, her identity as an experienced EGP teacher may make it especially difficult for her transition to an EAP teacher, a common challenge faced by many English teachers in China. Like many teachers in the EGP-EAP transition process, Lea felt disadvantaged in her EAP teacher identity formation and hence was involved in serious identity and emotional struggles. Second, Lea’s complete lack of leading experience may not only bring her tremendous identity and emotional tensions but also constantly push her to position and reposition herself in the fluid power relations with other stakeholders in the educational change. As a newcomer to the demanding EAP community together with her colleagues, Lea may find it very hard to attain her leadership authority and gain a central position in the reform community. Given that EAP teaching requires teachers’ academic literacy, Lea’s precarious power position may be further challenged by her previous MA training, which may well qualify her in a language-skill-focused EGP class but may seem rather inadequate in the community members’ eyes to build expert teacher identity in EAP teaching. Clearly, how could Lea who was under-prepared for the EAP teacher and teacher leader identities be able to cope with her emotional tensions, take agentic actions to lead the reform community, and achieve her power position in the workplace ecology deserves closer examination.

### Data collection and analysis

A case study methodology was adopted in this study for its merits in providing a rich, in-depth analysis of a complex social phenomenon, particularly over a period of time (Van Lier, 2005), with a focus on “the knowledge, performance, or perspectives of a single individual” (Duff, 2013, p. 1) in an “accessible, concrete, immediate, and personal manner” (Duff, 2012, p. 96).

Data were collected through ten rounds of semi-structured interviews spanning from June 2017 to December 2021 (Table 1) conducted over a half-year basis. For each semi-structured interview, Lea was inquired about her current impressions on the EAP reform, improvements made and difficulties encountered at the present stage, as well as views and suggestions on the prospects of the EAP reform. Questions varied across interviews based on Lea’s current situation at the time of the interview and her emergent responses to the interview questions. To avoid guiding her, great care was taken to ensure that she was free to express her thoughts without being interrupted. The interviews, conducted in Chinese, were audio-recorded, automatically transcribed by IFLYTEK intelligent recording pen, and checked word-for-word before being compiled into 139,506 Chinese characters. The selected interview excerpts were translated into English by Author 1 and doubled-checked by Author 2 until an agreement was reached. Emails and messages were exchanged between Lea and Author 1 for clarifications and further questions. The semi-structured interviews were triangulated by observations on group meetings for EAP textbook compiling, EAP lesson preparation meetings, several Lea’s EAP classes, and department-held share fairs on EAP teaching in 2019, 2020, and 2021. This paper primarily drew on the interview data with Lea.

**TABLE 1:** Timeline of data collection.

Interview	Time	Lea’s leading practices in the EAP reform
Round 1	June 2017	Preparing EAP textbook materials
Round 2	December 2017	Compiling EAP textbook
Round 3	June 2018	Revising EAP textbook and piloting teaching EAP classes
Round 4	December 2018	Large-scale EAP teaching at the university level
Round 5	June 2019	Publishing EAP textbook and preparing for EAP MOOC
Round 6	December 2019	Structuring EAP MOOC content and developing EAP tests
Round 7	June 2020	Collecting EAP MOOC materials
Round 8	December 2020	Making EAP MOOC
Round 9	June 2021	Making EAP MOOC and piloting teaching EAP MOOC
Round 10	December 2021	Teaching and moderating EAP MOOC and developing EAP tests

Ten interview transcriptions were read multiple times before they were analyzed in six steps (Table 2). Steps 2–6 were conducted separately by the two authors before they discussed and reached an agreement. Specifically, steps 1–3 targeted the research question about how emerging teacher leaders’ emotions change in the reform ecology; steps 3–5 attended to the research questions about the interplay of emotions, agency, and identities including emerging teacher leader identity in the reform process.

**TABLE 2:** Coding procedure.

**Step 1**	**Identify and classify emotional expressions**
	Affective expressions were identified and classified into positive and negative ones based on the National Taiwan University Sentiment Dictionary (NTUSD) (https://github.com/Leewin0821/text_mining) by using word EmEditor.
**Step 2**	**Filter irrelevant emotional expressions**
	Affective expressions that express factual statements were eliminated. For example, although the sentence *Writing the Introduction was not taught in the spring semester* contains *not*, a negatively affective word, it is factual rather than emotional and was thus excluded from further analysis. Affective expressions that are not associated with Lea’s own emotions were eliminated. For instance, although Lea said that *The students had many complaints*, the emotional expression *complaints* reflects her students’ emotions rather than her own and were thus excluded from further analysis.
**Step 3**	**Map emotional expressions onto different ecosystems**
	The screened positive and negative emotions were mapped onto different ecosystems within the reform ecology (i.e., microsystem, mesosystem, exosystem, macrosystem, and chronosystem).
**Step 4**	**Identify and categorize teacher identities**
	Reform-related teacher identities were identified and categorized into EGP teacher identity, EAP teacher identity, and teacher leader identity.
**Step 5**	**Identify and categorize agentic actions**
	Reform-related agentic actions were identified and categorized into different ecosystems within the reform ecology.
**Step 6**	**Formulate themes**
	Themes about the interactions of emotions, agency, and identities in different ecosystems within the workplace ecology were formulated.

## Findings

### Emotion and agency in reform ecology

Lea’s emotional changes in the EAP reform ecology generally exhibit two patterns. For one thing, as the reform advanced, Lea’s positive and negative emotions co-existed but generally declined; for another, Lea’s emotions showed an outward movement from inner systems (e.g., microsystem) to outer layers (e.g., exosystem) in the reform ecology. Both trends were made possible through her agentic actions taken at various levels of the reform ecology.

The first two rounds of interviews, in which the reform team’s main task was to compile an EAP textbook that helps graduate students write research papers, saw Lea’s intensive negative emotions because, at the beginning of reform, Lea was immersed with strong self-doubts and team members’ challenges regarding what to include in the textbook, whether EGP teachers are capable of teaching EAP knowledge, and whether she was competent for the leadership position in an EAP reform community. When describing her own feelings about the reform, Lea acknowledged:

Excerpt 1 (Round 1): With more than 20 years of EGP teaching experience, I found myself in complete lack of clear understandings about EAP teaching. I am aware that I am not entirely capable of the EAP classroom teaching, a great challenge full of both expectation and fear. Those academic skills are not only new knowledge to my students but new to me as well. I need to turn myself into a hardworking EAP learner before I could become a real EAP teacher.

Recognizing her own weakness in EAP knowledge and lack of academic literacy, Lea agentively turned herself into “a hardworking learner” who delved into the available EAP teaching resources (i.e., books, online lectures) to develop structural thinking of the textbook. Meanwhile, she experienced negative emotions toward the reform community because she had to promote its relational agency by “pushing all the teachers in the reform team to become EAP learners” (Round 2). However, given her equally non-expert teacher identity and less established authority position in the reform community, Lea’s acquired EAP knowledge did not sufficiently secure her leadership in the group.

Excerpt 2 (Round 2): There were several heated debates and even moments of crises when we discussed the materials collected for our EAP textbook. To many people, EAP reform is too hard a burden to bear for frontline teachers in China who are already heavily loaded in their teaching. I need to push all the teachers in the reform team to become EAP learners, which, I know, is hardly convincing to them because I myself is a learner.

As shown in Excerpts 1 and 2, before Round 4, most of Lea’s negative emotions were generated from herself and her colleagues in the inner systems, indicating her great emotional struggles in forming both an EAP teacher identity and even a teacher leader identity.

While capitalizing on her individual agency to construct her EAP teacher identity, she also consciously attended to the relational agency of the reform community. When encountered with severe emotional tensions between the reform-desired and her incompetent EAP teacher identity, she boosted the community’s relational agency to compose the EAP textbook by organizing regular weekly meetings for lesson preparation to equip the community members including herself with the necessary and “systematic” (Round 3) EAP knowledge before their EAP classroom teaching.

Excerpt 3 (Round 3): Teamwork is vital in efficiently and effectively dealing with the demanding EAP tasks. For individual teachers, it is almost impossible to acquire the complicated EAP knowledge on their own in such a short time. But in the group, we are able to split the learning tasks into much smaller pieces, with each one of us responsible for some manageable parts before the pieces of the EAP puzzle gradually gathered together to make the whole picture. I feel we are collaboratively creating this new world of EAP which is a still changing but pretty promising place.

As shown in Excerpt 3, Lea strategically scaffolded her community members with each other’s efforts in sharing the individually acquired EAP knowledge to promote relational agency among the reform community. However, despite such collaborative initiative, there had been disagreement among the community members on the teachability of EAP knowledge to their less proficient students, which caused severe emotional encounters between Lea and her cohort. The need to cope with her own emotional tensions and at the same time attend to her team members’ negative emotions gave rise to her agentic decision to implement a pilot teaching using their compiled textbook to a small scale of students in Round 3. Fortunately, the pilot teaching proved to be a great success and was well received among students, leading to her outnumbered positive emotions over negative ones in Round 3.

Excerpt 4 (Round 3): I was quite surprised when the students applauded at the end of my first EAP class. I thought they were just being polite. But later on I noticed they were actually concentrating on my teaching. Their eyes were on me almost all the time. When my second-week EAP class was over, I said to my students with all my sincerity: “Thank you very much for your attention.”

Impressively, the success of the pilot teaching also mitigated some of the community members’ negative emotions toward the EAP reform. Lea’s individual agency in pilot teaching reassured her community members’ concerns over the teachability of EAP knowledge to some extent and inspired them with students’ overall positive feedback to this course, somehow empowering the community’s relational agency to push the reform forward collectively. Clearly, Round 3 was a turning point for Lea’s emotional changes as she accumulated sufficient EAP knowledge in the textbook compiling process, built a certain level of self-confidence in her EAP literacy through pilot teaching, and, more importantly, “convinced” (Round 3) the community members that the EAP reform was a feasible and indispensable move.

As she constantly navigated the reform ecology, Lea developed more positive emotions toward her emerging EAP teacher identity. However, she did not explicitly identify herself as a teacher leader until Round 6 despite the fact she had been taking the leading position all along. Such acts of distancing herself from claiming the teacher leader identity suggest that her construction of leadership identity may involve constant and long-term emotional and identity struggles with both herself and her colleagues, which supports the Foucauldian position that power, though usually assigned, exists and evolves in specific social realities. Noteworthily, after Round 6, there was a general decline in both Lea’s positive and negative emotions despite the emotion-arousing emergency remote teaching caused by the pandemic in Spring 2020 (i.e., Round 7). This can be seen in light of Gao and Cui’s (2021b) finding that as a teacher leader established sufficient reform-required knowledge and gained authority in the reform community, s/he tended to be involved in fewer emotional tensions and struggles. Thus, the overall reduced positive and negative emotions indicated Lea’s established EAP teacher and emerging teacher leader identities. That is, it was Lea’s self-recognition as an EAP teacher leader and a confident EAP teacher that contributed to her less drastic emotional fluctuations during the educational reform. Interestingly, with her established teacher leader identity, Lea targeted some of her emotions, mostly negative, toward more stakeholders of the educational reform (Gao and Cui, 2021b), which resulted in her overall reduced but continuous emotional investments. These newly emerged emotions prompted her further agentic actions.

One such emotional target involved the university’s English policy, an outer layer (i.e., exosystem) in the reform ecology, which brought her intensive negative emotions. The EAP course was designed to be delivered in two academic semesters, with the fall semester primarily focusing on developing the Introduction of a research paper by paraphrasing, summarizing, and synthesizing selected literature, and the spring semester mainly devoted to describing research methods, reporting and discussing results using appropriate academic language. However, the university policy allows graduate students to receive credit for English course within one semester (i.e., often the fall semester) if they pass a final exam so as to spend the rest of their school years entirely concentrating on their academic research. Such a policy leaves a good number of students having no access to the full content of the EAP course and unable to develop a “systematic” (Round 4) understanding of EAP knowledge, which made students “care too much about their final grades” (Round 4) and too little about what they could truly learn in the EAP classes.

Excerpt 5 (Round 9): The Fall 2018 university-level EAP teaching experience was special to me because I realized then that our university policy about the final exam may have an adverse effect on our EAP teaching outcome and prevent our reform efforts from achieving its full potential.

As shown, Lea’s negative emotions underwent a gradual outward extension, firstly arising mainly from her interactions with herself and colleagues in the micro- and meso-systems and later with such organizations as the university in the exosystem. To counter such policy imposed on their EAP teaching, Lea agentively proposed to make an EAP MOOC that covers the whole textbook in 90 mini-lectures. Clearly, her agentic move to make an EAP MOOC not only originated from the limited online teaching resources available to accommodate the emergency remote teaching during the pandemic and was encouraged by her increasingly positive emotions toward herself as an EAP teacher and teacher leader in the mesosystem, but also aimed at contesting the English teaching policy in her university.

Notably, Lea took agentic actions as her active responses to her emotional tensions. Her intense emotions were, indeed, valuable sources of her devoted engagement and painstaking efforts in coping with the major conflicts in the reform ecology, because her emotional stresses largely arose from the great discrepancy between the reform reality and the reform requirements, and hence served as her agentive guide and critical strategy to overcome the practical problems and achieve the reform goals, further reflecting her negotiation of a teacher leader identity.

Noteworthily, Lea took cross-ecosystem agentic actions to reconcile her emotional tensions. When running into emotional challenges with herself due to the clash between EGP and EAP identities in the microsystem, she exercised her agency in the mesosystem with her colleagues to co-construct the knowledge system necessary for EAP teaching; when coming across emotional disputes with her colleagues in the mesosystem for their conflicting beliefs about the EAP reform, she practiced her agency by piloting a trial teaching with her students in the microsystem, a fairly controllable teaching experiment considering the small number of student participants compared with the whole graduate student population in the university. Lea strategically wove her way to cope with her emotional difficulties, rather than directly confronting them by performing agentic actions immediately against the real sources of her negative emotions.

### Agency and power in reform ecology

Lea’s roundabout cross-ecosystem agentic actions served as effective strategies to build up her strong power positions in her relations with herself, her students, and her colleagues. Lea achieved a sense of control as an EAP teacher and a sense of authority over her students in the microsystem and attained the leadership position of the reform community in the mesosystem by playing the role of an active agent who sought support from different sources.

Lea’s agentic actions with her colleagues in the mesosystem to compile the EAP textbook and prepare EAP lessons qualified her for a “confident” EAP teacher identity which grew into the dominant part of herself as an English teacher despite her 20 years of EGP teaching experience and hence solved the uneasy power relations between her incompatible identities. Her EAP classroom teaching shaped her self-image as a “guide” (Rounds 7 and 8) and her students ideally as “close followers” (Round 8) who were not “particularly interested in EGP classes” (Round 3) and whose academic development could “certainly benefit from the EAP lessons” (Round 5) regardless of EAP teachers’ deficit disciplinary knowledge in students’ specific research areas. Lea viewed her ability to “directly address students’ academic concerns” as “a sense of self-worth” (Round 3).

More importantly, Lea’s pilot interactions with students in classroom teaching in Round 3 formed her more of the expert teacher identity than those colleagues resisting EAP teaching and gained her authority to lead the reform community through the educational change. Unlike at the beginning of the EAP reform when everyone was new in the community, from Round 4 onward, Lea has been taking the power position to assess and even criticize some of her colleague’s EGP-like teaching in their EAP classes, before formally claiming herself as a teacher leader in Round 6.

Excerpt 6 (Round 6): Some of our teachers are in serious need to improve their understanding about EAP teaching and the related EAP knowledge. They are still confused about the role English teachers could possibly play for science students. They may have very little idea about the genre features of academic texts. For example, they teach paraphrasing and summarizing without realizing their importance in academic writing. Simply staying on the superficial level of the knowledge points, they failed to form a logic line that underlies research paper writing.

Lea’s criticality toward her colleagues’ less satisfactory teaching indicates her established EAP teacher identity and emerging teacher leader identity who starts to focus more on the relational agency within the reform community. However, Lea’s cross-ecosystem agentic actions may ease her power tensions and win her full power in some layers of the workplace ecology, but only partial power in others, as shown in her power interplay with the university at the exosystem. As previously discussed, Lea has been engaged in constant negotiations with the university since Round 4 when she realized that the university’s credit system could strongly impact the reform outcome. Her agentic reaction to contest the university’s “unfriendly” (Round 5) policy was seen in her “eager and determined” attitude and preliminary plan to make a MOOC with her colleagues in the mesosystem as a convenient supplement to possibly make up for the missing EAP part and even to innovate the current EAP teaching by adopting a blended teaching mode that combines offline classroom teaching with online resources.

Lea’s online–offline blended teaching plan seemed to accelerate during the COVID-19 pandemic when distance learning was promoted as the main educational tool. On the one hand, there was an impetus to invest in making online courses on the national scale considering the very limited remote teaching resources; on the other hand, despite its determination to support Lea’s MOOC plan, the university’s teaching affairs office needed a long time to choose the online teaching platform and negotiate with the MOOC-producing company. For all Lea’s yearning desire to reach out for technical details that will assist different online teaching possibilities, she found herself powerless in seeking immediate and substantive support beyond campus before any formal financial agreements can be made at the university level. Again, rather than keeping struggling with the external companies in the exosystem, she took active action with her colleagues in the mesosystem (Excerpt 7) and her students in the microsystem (Excerpt 8) instead.

Excerpt 7 (Round 7): Our pre-MOOC group met every week to discuss the important EAP knowledge points to be included in the mini-lectures for our next-step screen recording. For each 10-min mini-lecture, one teacher was responsible to prepare the power point and the word-by-word script, and polish them over and over again based on the comments and suggestions from others in the group. Our initial preparation work ensured efficient and smooth cooperation afterward when we were given a green light to make MOOC.

Excerpt 8 (Round 8): To consider students’ feedback on our MOOC lectures exclusively delivered in English, we presented our self-made mini-lectures in our offline classes to see whether our students were truly interested in those knowledge points and whether our speaking rates were natural and normal to them. We also wanted to decide if we need to include captions or not.

Lea’s hope for blended teaching was dashed when the teaching affairs office made it clear that the university curriculum would include either online or offline EAP courses as opposed to a blended one at the same time. However, with Lea’s efforts, her department (i.e., mesosystem) and the teaching affairs office (i.e., exosystem) collectively modified the English policy so that the EAP MOOC can cover a wide student population on campus, with roughly 3,000 students enrolled each semester. This large number though benefited more graduate students than Lea hoped, highly restricted teacher-student interactions. In such a “frustrating” (Round 9) MOOC teaching reality structured by the university policy in the exosystem, Lea took agentic actions and directly targeted the students in the microsystem by engaging them in the course discussion board where communication was afforded by the digital space (Excerpt 9) and by organizing regularly held extracurricular teacher–student offline seminars (Excerpt 10).

Excerpt 9 (Round 10): It seems that once the EAP MOOC was online, students could learn on their own whereas teachers’ part to play was done. That basically put our traditional role and responsibilities as teachers much into question. I found the Discussion Forum on the MOOC platform could be our useful interactive territory, where we could lead discussions to make our online students visible. Every week, I put up a discussion topic related to the MOOC content. And for every topic, more than 300 students took part in the teacher-student or student-student discussions.

Excerpt 10 (Round 10): Last Wednesday, MOOC teachers held a Q & A offline meeting with students. Sixty students participated and raised a variety of questions about their difficulties in MOOC learning. For example, students may find it hard to understand a particular knowledge point. We then took the opportunity to explain to them how different knowledge points are closely connected rather than randomly scattered as presented in the MOOC and how different knowledge points could work together to contribute to paper writing. I think it is valuable to have such communications. I was very excited.

In summary, the cross-level agency Lea exercised to counter her negative emotions experienced at various levels prompted her agentic actions across the reform ecology and gradually gained her certain central power in the reform community. Not surprisingly, in her exercise of agency, more emotions might arise but may be leveraged through taking further agentic actions. Noteworthily, in such an ongoing negotiation of power and identity in the highly dynamic and evolving educational reform context, it is the intricate interrelationship between emotion, agency, and power that progressively empowered Lea for upward leadership mobility in the reform ecology.

### Power and emotion in reform ecology

Lea’s power status within a workplace ecological system was a strong indicator of her emotional experiences in that particular ecosystem. For instance, Lea’s power negotiations with the university in the exosystem since Round 4, though obtained some control and authority through her agentic actions in the micro- and meso-systems, failed to build her a solid power position to implement her plans for blended teaching, and thus caused many of her negative emotions. Her emotional tensions with the university displayed ups and downs because her power relations with the university alternated between a “helplessly” (Round 9) problematic situation and a hopeful future in which she was able to “make some difference” (Round 10). Lea’s seesaw power conflicts with the university were shown, on the one hand, in her emotionally out-of-control moments when she complained about the university’s credit system that “ruined” (Round 6) the fall semester’s EAP teaching outcomes in Rounds 4 and 6 and when she felt at a loss for the university’s dismissal of her blended teaching proposal in Round 9 and were demonstrated, on the other hand, in her calm-down intervals when she seized some sense of power to improve the unfavorable credit condition by planning and making MOOC in Rounds 7 and 8 and to create chances to form connections with MOOC students in Round 10, as previously discussed.

The power relation in an ecosystem gave rise to the dynamic fluctuations of Lea’s emotions toward the stakeholder in that power relation. For example, MOOC as a mode of teaching that is different from the traditional in-person classroom instruction redefined the teacher-student power relation and thus reshaped Lea’s emotional experiences with students. Unlike in face-to-face classrooms where she possessed the power to “guide” (Rounds 7 and 8) her students, she found herself much reduced to a lesson deliverer and course administrator instead of a respectable and admirable teacher on the MOOC platform, which resulted in the increase of her negative emotions against the MOOC students in Round 9. Given a large number of students and a shortage of available instructors to offer immediate feedback on student assignments, Lea came up with the idea of doing a peer review in which each student was given a grading rubric and assigned five other students’ answers to grade, and one student’s final score on certain assignment was an average of five students’ ratings. Unexpectedly, such a rational decision was seriously complained about by students who received “irresponsible and random scores” (comment from a MOOC student). As seen in Excerpt 11, Lea’s unpleasant engagement with MOOC students’ complaints and challenges negatively affected her self-recognition as an established EAP teacher and teacher leader identity.

Excerpt 11 (Round 9): There were nearly 3,000 MOOC students this semester. Our weak voices were drowned in the flood of their endless complaints and challenges irrelevant to the EAP course itself, many of which could be very rude. … It was shocking to see many of the MOOC students turned into keyboard heroes. It was even shocking that I found myself as a highly appreciated teacher involved in this tug of war with students in fear that my home field was taken over.

Similarly, the power relation in the reform community went through a dynamic change which outlined Lea’s fluctuating emotional experiences with her colleagues in the mesosystem. In Rounds 1 and 2, Lea was exposed to many emotional tensions with her colleagues who questioned her leading power in the reform community because of her yet acquired identities as an EAP teacher and a teacher leader. In Rounds 3 and 4, through the success of EAP pilot teaching, Lea gradually developed her EAP teacher identity and built up her central power position in the reform community, which considerably relaxed her emotional tensions with her colleagues. In the subsequent rounds, however, Lea’s emotional conflicts with her colleagues occurred from time to time because new teachers without any previous EAP teaching experience kept joining the reform team as a result of faculty shortage and somehow disturbed the power relation that evolved in the first few stages of the reform. Different from the early reform participants who were motivated to make the challenging EGP-EAP transition collectively and gradually endorsed Lea’s power position in the community through her efforts to promote individual and relational agency, later people who were comparatively less compliant to the central power and more independent from the community leader and other members made the reform community a less collaborative whole, and hence the community’s relational agency was somehow reduced. Such non-conformity and lack of “hot blood” (Round 6) among new members also profiled Lea’s negative emotions after Round 6.

Excerpt 12 (Round 6): The early days of our reform are the time of hot blood when we had heated debates on our opposing beliefs and nevertheless huddled close to fight this tough war against our fear toward EAP. I am now worried about newcomers to our reform community including foreign instructors with no EAP background, Chinese English teachers experienced in EGP teaching, and novice teachers with little teaching experience, who walked right into EAP classrooms with their own EAP understandings and without going through the struggling process of collective self-training as we did. I feel they are the reform outsider.

To actively engage and motivate these teachers, Lea again resorted to her individual agency to strategically initiate group collaborations such as designing EAP tests; however, these efforts failed to create “sincere thoughts and excellent ideas” that may “clash with others’ but were highly appreciable” (Round 10), which made her feel nostalgic about “the old days” (Round 10). It is evident that Lea’s established power in the reform ecology urged her to attend to other members’ participation in the reform, which may cause additional emotional tensions. On the other hand, her gained central power position in the reform community entailed her pride and confidence in their EAP reform which has “a bright future” and is “exerting its powerful impact in China’s EAP teaching field,” and boosted her determination to “maintain the high EAP teaching standard” (Round 10), which calls for every group member’s contributing efforts. The negotiation of these mixed power-generating emotions further reinforced Lea’s power position in the reform ecology.

## Discussion

By longitudinally tracing an EAP teacher and at the same time a teacher leader in an educational change context, the present case study shows that emotion, agency, and power are all multilayered, highly dynamic constructs in an educational reform context. In line with previous studies (Cross and Hong, 2012; Chen, 2017; Gao and Cui, 2021b; Hsieh et al., 2022) that situated teacher identities and teacher emotions in multiple subsystems of workplace ecology, our study on Lea’s case further points out that teachers in an educational change take different agentic actions (Ketelaar et al., 2012; Pyhältö et al., 2012; Dover et al., 2016; Ruan et al., 2020) and practice different powers (Zembylas, 2005; Benesch, 2018) in the multi-leveled ecosystems in relations with their identities and emotions, which makes teacher development an exceedingly complex process. Rather than viewing teacher emotion, agency, and power in professional development as changes in a simple trajectory, our study peels up the reform ecology to gain fresh insight into the changes in the moving wheels of reform ecosystems that evolve not necessarily at the same pace. Individual teachers’ active interactions with different ecosystems in the reform environment make workplace ecology an intricate dynamic that, if vaguely portrayed, may not present a clear and complete picture of teacher development. In future studies, therefore, more research efforts are called for to examine teacher emotion, agency, and power, and their changes and interplays with respect to specific stakeholders in certain ecological systems to form a fine-grained understanding of teacher development.

What makes the dynamic change of teacher development even more complex is that individual teachers’ emotions, agency, and power relations in one subsystem may influence those constructs in others. Our findings based on Lea’s case echo previous studies (Darling-Hammond et al., 1995; Zhang and Henderson, 2018) in that teacher leaders’ power position in one ecosystem may strengthen their power hierarchy in another. In particular, our study advances the extant literature by elaborating on the cross-ecosystem influence of teacher leader agency upon leadership power in that emerging leaders’ agentic actions in one ecosystem may help build up their authority and identity in another. Instead of directly acting against the power conflicts in an ecosystem, emerging teacher leaders like Lea may actively seek possible power support from external resources in the reform ecology. Our findings are in accordance with the studies (Elfenbein and Ambaby, 2002; Wu and Chen, 2018) that highlighted the cultural-specific feature of workplace power since the indirect practice of teacher leader agency may pertain to the Chinese cultural tradition that characterizes indirectness as the dominant style of interpersonal communications (Yeung, 2000), especially in subordinate-superior interaction scenarios. Considering the emerging teacher leaders’ leader-to-be relation with their colleagues and subordinate power position with the higher-level ecosystem, it might not be wise for them to take the upper-hand role which may cause direct confrontations that could seriously hold back the educational reform, and it might be more realistic for them to get around the existing power conflicts in order to promote the reform in the most efficient way.

Our study confirms the important role of individual agency in teacher development (Ketelaar et al., 2012; Pyhältö et al., 2012; Dover et al., 2016; Huy et al., 2016; Ruan et al., 2020). We also highlight the critical role of an emerging teacher leader’s individual agency to promote the relational agency of the reform community by convincing community members to make joint efforts, pursue a common interest, and shape a shared future (Hökkä et al., 2017). Such agentic actions are in an agreement with the collectivist cultural tradition (Hofstede, 2011) that follows the Confucian epitome to honor seniority and authority as widely recognized in workplace settings in China. This usually results in highly valued centralized power positions (Sundararajan, 2015) as is shown in Lea’s case which also proves how the cultural values in the macrosystem as abstract institutions may permeate their far-reaching and long-lasting effects on the inner systems in the workplace ecology (Wu and Chen, 2018). By boosting the relational agency in the reform community to collaboratively compile an EAP textbook, Lea managed to equip herself and her colleagues with the necessary EAP knowledge, which gained her power in the EAP classroom with her students to build her EAP teacher identity, which, in turn, convinced her colleagues about the practicality of EAP reform so as to attain her leadership power in the reform community. The emerging leader’s individual agency to enhance relational agency in the reform community, on the one hand, rapidly carried forward the educational reform process, and on the other hand, contributed to a certain level of achievements (Friesen and Besley, 2013) of both the reform-required teacher identity and teacher leader identity.

Like previous conceptualizations of identity as a dynamic and ongoing construct (e.g., Thomas and Beauchamp, 2011; Taylor, 2017; Tao and Gao, 2018), power is also co-constructed in situated social relations. In an evolving educational reform ecology that involves multiple stakeholders at various levels, although Lea’s agentic actions at some ecological level may effectively reconcile her emotional struggles and promote her power negotiations within that system, the intricate interrelationship between emotions, agency, and identity makes the ultimate achievement of power a continuing effort. Specifically, in Lea’s case, her established teacher leader identity may only be secured at the micro- and meso-systems, with her power at the exosystem and even the macrosystem largely marginalized. Nevertheless, her ongoing negotiations of emotions, agency, and identity may gain her upward power mobility that may trigger further emotions, agentic actions, and identity achievements. In a word, Lea’s power status in the reform ecology should be seen in light of the Foucauldian conceptualization that power is co-constructed and constantly evolves in social realities.

**FIGURE 1:**
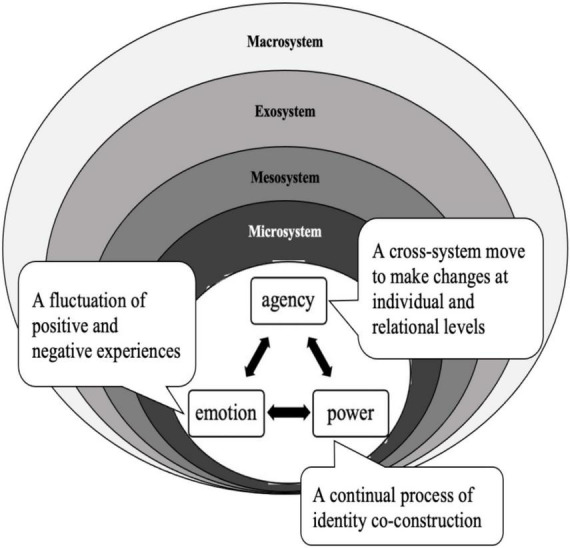
Emerging teacher leaders’ emotion, agency, and power in reform ecology.

Confirming the crucial role of teacher leadership in educational reform contexts (Harris and Muijs, 2003; Fullan, 2005), our case study further clarifies that leadership power could be negotiated through emerging teacher leader’s agentic actions, and the change of power relation could accordingly influence the dynamic development of teacher emotions in the reform ecology. On the one hand, the sense of powerlessness in identity construction in an educational reform may lead to enormous emotional struggles (Akkerman and Meijer, 2011; Arvaja, 2018; Gao and Cui, 2021a,b), and regaining the controlling power may greatly reconcile emotional tensions. On the other hand, as emotional tensions relaxed at a certain ecological level, teacher leaders may lose the driving force to take intentional actions against the reform problems in that ecosystem. Rather than passively waiting for the advent of leadership power, emerging teacher leaders should exercise their agency to respond strategically and act decisively (Ketelaar et al., 2012; Pyhältö et al., 2012; Dover et al., 2016) to cope with the demanding reform situation and have their powers emerged and their emotional difficulties eased along the way. In contrast with previous studies (Zembylas, 2004, 2005; Benesch, 2017, 2018) that focused on the unidirectional impact of power position upon teacher emotions and teacher agency, our research on Lea’s case sheds light on the mutually interrelated connections among emotion, agency, and power by highlighting the importance of teacher leaders’ agentic actions in their identity and power construction and the consequent emotional change. Therefore, we propose a model for emerging teacher leaders (Figure 1), indicating the intimate ties of emotion, agency, and power in a reform ecology and their respective cross-level influence on various reform participators, which may also bear implications for not just emerging teacher leaders but also teacher leaders and the general teacher population in an educational reform.

## Conclusion

By situating Lea’s emotional responses to the educational reforms in the 5-year timespan, this study reveals how a reform-inflected emotional changes were associated with her EAP teacher and teacher leader identity construction. It is Lea’s emotional tensions experienced across the different layers of the reform ecology that motivate her agentic actions to gain her leadership power in the reform community and contribute to her construction of the EAP teacher identity upon which her teacher leader identity also emerged, which to some extent reconciled her emotional tensions. By presenting the intricate interrelationship between teacher emotion, agency, and power, we highlight that LTP is a dynamic construct that needs to be examined in a larger timescale and broader educational context to advance LTP research and to benefit language teachers’ professional development. Most importantly, our proposed model may shed light on emerging teacher leaders’ emotion, agency, and power in reform ecology, motivating further research with different teacher groups and various levels of educational institutions.

Although we focused only on one participant in this case study, which may restrict the generalizability of our findings, the longitudinal and triangulated data enriched the thick description of our case, making the implications significant. For language teachers involved in an educational reform, individual agentic actions at the microsystem (e.g., building the new knowledge) and within-community collaborations at the mesosystem may create space for teachers’ identity construction and thus professional development; for teacher leaders, especially emerging teacher leaders, cross-level agentic actions may counter reform-inflected emotional tensions generated in different ecosystems; such a strategic detour may also avoid direct confrontations with the reform stakeholders and transform teacher agency into powerful leadership in the reform ecology; for school administrators at the exosystem, reform participators’ psychological traits including their emotions should be considered as vitally important in a demanding reform ecology, and their agentic actions should be greatly embraced in different workplace ecosystems to ensure the success of an educational reform that conforms to educational policy initiated at the national macrosystem.

## Data availability statement

The raw data supporting the conclusions of this article will be made available by the authors, without undue reservation.

## Ethics statement

Ethical review and approval was not required for the study on human participants in accordance with the local legislation and institutional requirements. The patients/participants provided their written informed consent to participate in this study. Written informed consent was obtained from the individual(s) for the publication of any potentially identifiable images or data included in this article.

## Author contributions

YG: conceptualization, methodology, data curation, investigation, formal analysis, software, visualization, writing – original draft, and project administration. YC: conceptualization, methodology, investigation, formal analysis, and writing – original draft. Both authors contributed to the article and approved the submitted version.
